# Anxiety, Post-Traumatic Stress, and Burnout in Health Professionals during the COVID-19 Pandemic: Comparing Mental Health Professionals and Other Healthcare Workers

**DOI:** 10.3390/healthcare9060635

**Published:** 2021-05-27

**Authors:** Isabella Giulia Franzoi, Antonella Granieri, Maria Domenica Sauta, Monica Agnesone, Marco Gonella, Roberto Cavallo, Piergiorgio Lochner, Nicola Luigi Bragazzi, Andrea Naldi

**Affiliations:** 1 Department of Psychology, University of Turin, 10124 Turin, Italy; antonella.granieri@unito.it (A.G.); mariadomenica.sauta@unito.it (M.D.S.); marco.gonella@unito.it (M.G.); 2 SS Psychology, Local Health Authority “Città di Torino”, 10143 Turin, Italy; monica.agnesone@aslcittaditorino.it; 3 Neurology Unit, San Giovanni Bosco Hospital, 10154 Turin, Italy; roberto.cavallo@aslcittaditorino.it (R.C.); naldi.andrea@yahoo.it (A.N.); 4 Department of Neurology, Saarland University Medical Center, 66421 Homburg, Germany; piergiorgio.lochner@gmail.com; 5 Laboratory for Industrial and Applied Mathematics (LIAM), Department of Mathematics and Statistics, York University, Toronto, ON M3J 1P3, Canada; robertobragazzi@gmail.com; 6 Department of Neuroscience “Rita Levi Montalcini”, University of Turin, 10126 Turin, Italy

**Keywords:** COVID-19, mental health, mental health professionals, pandemic, distress, risk-factors, protective factors

## Abstract

The psychological impact of the pandemic on healthcare workers has been assessed worldwide, but there are limited data on how mental health professionals (MHPs) have been affected. Thus, this paper aims to investigate anxiety, post-traumatic stress, and burnout in a sample of MHPs. We conducted a descriptive, cross-sectional study on 167 participants: 56 MHPs, 57 physicians working closely with COVID-19 patients, and 54 physicians not working closely with such patients. MHPs reported good overall mental health. Most MHPs reported no post-traumatic stress, and their scores were significantly lower compared to HPs working closely with COVID-19 patients. MHPs’ hyperarousal scores were also significantly lower compared to HPs working closely with COVID-19 patients, while their intrusion scores were statistically significantly lower than those of all other professionals. Multivariable logistic regressions showed that MHPs had lower odds of exhibiting state anxiety and low personal accomplishment compared to HPs not working closely with COVID-19 patients. In sum, MHPs seem to show almost preserved mental health. Thus, given the high mental healthcare demand during a pandemic, it would be useful to rely on these professionals, especially for structuring interventions to improve and support the mental health of the general population and other healthcare workers.

## 1. Introduction

Since the novel coronavirus disease (COVID-19) emerged in China at the end of 2019 and was subsequently classified as a pandemic by the World Health Organization on 11 March 2020, its impact on society has been enormous. Worldwide, authorities have had to take extreme actions to contain its spread. In the initial absence of a vaccine, quarantine has been implemented as one of the most effective measures to reduce transmission risk. The disease itself, fear of contagion, and the different containment measures have significantly affected people’s occupational and social lives, as well as their physical health and psychological wellbeing [1]. A pandemic can place severe strain on general mental health resources, potentially leading to untreated mental health issues [2,3,4]. A large range of psychological outcomes emerged during the virus outbreak and continue to be experienced: stress, anxiety, depression, and fear of getting sick or dying [5,6]. Moreover, symptoms of post-traumatic stress disorder or complicated grief disorder have emerged, as they often do in the aftermath of global emergencies or disasters [7,8,9,10,11]. Prolonged social distancing, social isolation, and the illness or loss of family members can substantially exacerbate mental health issues [5,12,13,14,15,16]. In particular, recent studies show that people held in isolation or quarantine experienced significant levels of anxiety, anger, confusion, and stress [17,18]. Moreover, fear of infection can cause pervasive and persistent worries over one’s health and the potential risk of infecting others, especially loved ones [19,20].

Although a pandemic affects the entire population, individuals that are most exposed have a higher risk of developing mental health problems [21,22]. Research conducted during the outbreak of MERS and SARS showed that healthcare workers (HCWs) had the greatest risk of experiencing psychological distress [23]. The feeling of uncertainty, threat to health, and somatic symptoms were particularly prevalent [24]. The psychological impact of the COVID-19 pandemic on frontline HCWs has been assessed worldwide through numerous studies. During the SARS-CoV-2 pandemic, HCWs are facing aggravated psychological pressure and seem to be more severely affected by mental health issues [25,26,27] and indirect traumatization [21] than other occupational groups. In particular, they seem to experience depression, anxiety, traumatic stress, avoidance, and burnout [28]. Depression and anxiety symptoms reported by HCWs range from moderate to severe; moreover, the literature reveals that anxiety is positively correlated with the total amount of stress and workload [29,30]. Isolation from relatives, working in high-risk wards, fear, and feeling guilt over contributing to contagion have all been reported as major causes of trauma [31].

However, it seems that too little attention has been paid to the pandemic’s impact on mental health professionals (MHPs); to date, only Joshi and Sharma [32] have preliminarily underlined those stressors that put MHPs at risk of burnout, such as staff shortage and workload increase. Therefore, how the pandemic has affected the psychological wellbeing of MHPs remains largely unknown.

Thus, this paper investigates anxiety, post-traumatic distress, and burnout in a sample of MHPs, comparing their distress to that experienced by other health professionals (HPs), including those working closely and those not working closely with COVID-19 patients. We aim to detect potential sociodemographic, family, and occupational factors influencing the emotional impact of the pandemic.

## 2. Materials and Methods

### 2.1. Study Design and Participants

We conducted a descriptive, cross-sectional study through a web-based survey. Enrollment progressively included HCWs of four hospitals in Turin, Piedmont, North Italy, all directly engaged in managing COVID-19 patients.

The survey was sent by email to all HCWs of the four hospitals on 27 April 2020, explaining that study recruitment would end at midnight of the day on which the target sample size was achieved. The minimum sample size (*n* = 377) was calculated through the formula *N* = *Z*^2^ × *p*(1 − *p*)/*α*^2^ (considering a margin of error of 5% (*α* = 0.05), a 95% confidence interval (*Z* = 1.96), and a population proportion of 50% (*p* = 0.50)). However, to ensure meaningful subgroup analysis, the target sample size was doubled. Hence, at midnight of 1 May 2020, the survey was closed after a total of 962 HCWs had completed the questionnaire. This study focused on the experience of MHPs (including psychologists and psychiatrists, *n* = 56), compared with that of physicians who worked closely with COVID-19 patients receiving high-intensity care (*n* = 57) and physicians who reported none or only occasional contact with COVID-19 patients receiving low-intensity care (*n* = 54). Details of the different specializations of participating physicians are provided in Table 1. Psychological data regarding the entire population of physicians and nurses have previously been published [30].

Recruitment was undertaken in an especially demanding period of the COVID-19 health crisis in Italy: from March 10 to 3 May 2020, a nationwide quarantine was imposed, and the total numbers of COVID-19-confirmed cases exceeded 26,000 in Piedmont and 207,000 in Italy [33].

### 2.2. Survey Instrument

A web-based survey was developed to investigate burnout, anxiety, and post-traumatic stress in a sample of HCWs during the COVID-19-related health emergency in Italy, and to detect potential sociodemographic, family, and occupational factors. We chose this data collection method to ensure social distancing, as required by the Italian government at the time of the enrollment. The questionnaire was completed anonymously and administrated in Italian. The survey was conducted through Google Forms, a survey-administration app in the Google Drive office suite.

The first page of the web survey provided study details and required all participants to give informed consent by ticking the box marked “agree” before being allowed to access the next page. The survey then investigated sociodemographic, family, working, and psychological characteristics of the recruited sample. Sociodemographic data included age range, sex, and working position. Family data included having children, living situation before the pandemic (with or without partner), and occurrence of family division due to COVID-19 for safety reasons and/or fear of contagion. Working data included contact frequency with COVID-19 patients (frequent, rare, or none), the extent of work changes related to the pandemic, and changes in the relationship with patients (improved, worsened, or unchanged). Finally, psychological data were investigated through a pool of self-report measures assessing anxiety, post-traumatic stress, and occupational burnout, previously validated for the Italian population.

### 2.3. Outcome Measures

The self-report psychological measures comprised the State-Trait Anxiety Inventory-Y (STAI-Y) [34,35], the Impact of Event Scale-Revised (IES-R) [36,37], and the Maslach Burnout Inventory (MBI) [38,39].

The STAI-Y is a self-report inventory aimed at assessing both state (STAI-Y1) and trait (STAI-Y2) anxiety. State anxiety is the feeling of insecurity and helplessness in the face of a perceived threat that may lead to concern or avoidance, while trait anxiety is the tendency to experience and report, across many situations, negative emotions such as fears, worries, and anxiety. Each dimension comprises 20 non-overlapping questions. Participants are asked to rate how accurately each item describes them on a four-point scale (1 = “almost never” to 4 = “almost always”). Total scores for both scales range from 20 to 80. Consistent with previous research [40,41], we used a cut-off of 40 to evaluate the presence or absence of state and trait anxiety.

The IES-R is widely used to measure distress symptoms connected to traumatic events. It comprises 22 items assessing symptoms of avoidance (i.e., keeping away from particular activities, thoughts, etc. because of their anticipated negative consequences), intrusion (i.e., inability to keep memories of the event from returning), and hyperarousal (i.e., tendency to be easily startled, constant feeling that danger or disaster is nearby, inability to concentrate, irritability, or even violent behavior). Participants are asked to rate how distressing each item has been during the past week on a five-point Likert scale (0 = “not at all” to 4 = “extremely”). The total score ranges from 0 to 88, with a score of 33 or higher indicating possible presence of post-traumatic symptoms [42]. In particular, 0–23 indicates the absence of post-traumatic symptomatology, 24–32 indicates clinically detectable post-traumatic symptomatology, 33–36 indicates the possible presence of post-traumatic symptoms, and ≥37 indicates severe post-traumatic symptomatology.

The MBI is a measure of occupational burnout comprising three scales: emotional exhaustion (EE, nine items), examining the feeling of being emotionally bitter and exhausted from work; depersonalization (DP, five items), which measures a cold and impersonal response; personal accomplishment (PA, eight items), assessing the feeling of own competence and desire to succeed in working with others. High EE and DP scores indicate a higher level of burnout, while high PA scores indicate a lower level of burnout. Participants are asked to rate how often they experience what each item describes on a seven-point Likert scale (0 = “never” to 6 = “daily”). Consistent with previous research [43,44,45], we considered the level of burnout to be high with EE ≥ 24, PA ≤ 29, and DP ≥ 9, moderate with EE = 15–23, PA = 30–36, and DP = 4–8, and low with EE ≤ 14, PA ≥ 37, and DP ≤ 3.

### 2.4. Statistical Analysis

Data analyses were conducted using the Statistical Package for the Social Sciences (SPSS; IBM Corp., Armonk, NY, USA) version 26. We first computed skewness and kurtosis, and tested for normality with a Kolmogorov–Smirnov test. Since no continuous variables were normally distributed, they were all expressed as median and interquartile range (IQR) values, while categorical variables were expressed as frequency and percentage values. For continuous variables, differences between independent groups were evaluated using a Kruskal–Wallis H test. Subsequently, pairwise comparisons were performed using Dunn’s [46] procedure with Bonferroni correction for multiple comparisons. Categorical variables were compared using a chi-square test. Post-hoc analyses with Bonferroni corrections were conducted using adjusted residuals. Then, five multivariable logistic regressions were performed to ascertain the effects of possible risk factors on the likelihood of showing state anxiety, moderate-to-severe post-traumatic symptoms, high emotional exhaustion, high depersonalization, and low personal accomplishment. All tests were two-tailed, and we set the statistical significance threshold at *p* ≤ 0.05.

## 3. Results

### 3.1. Participants

As reported in Table 1, the final sample comprised 167 participants. Among the 56 MHPs, 40 (71.43%) were psychologists and 16 (28.57%) were psychiatrists. Among the 57 physicians working closely with COVID-19 patients, 28 (49.12%) were anesthesiologists, 15 (26.32%) were infectious diseases specialists, and 14 (24.56%) were ER physicians. The 54 physicians not working closely with COVID-19 patients had various specializations: for example, eight (14.81%) were preventive medicine specialists and seven (12.96%) were endocrinologists.

As Table 2 shows, no MHPs reported frequent contact with COVID-19 patients.

As reported in Table 3, most MHPs were female (48, 85.71%) and aged ≥41 years (50, 89.28%).

No interactions were found between professional groups and gender, but there was a statistically significant association between professional groups and age range, *χ^2^*(4) = 43.52, *p* < 0.001. This association was moderately strong [47], Cramer’s *V* = 0.361. In particular, post-hoc analysis using adjusted residuals demonstrated that MHPs were significantly less likely to be in the ≤40 years group (*z* = −3.05; *p* = 0.021) and more likely to be in the 41–50 years group (*z* = 3.12; *p* = 0.016), while HPs not working closely with COVID-19 patients were more likely to be in the ≥50 years group (*z* = 4.36; *p* < 0.001). Moreover, HPs working closely with COVID-19 patients were significantly more likely to be in the ≤40 years group (*z* = 5.52; *p* < 0.001) and less likely to be in the ≥50 years group (*z* = −4.04; *p* = 0.001).

### 3.2. Family Conditions

As Table 3 shows, most MHPs reported having children (37, 66.07%). The majority were not living alone before the pandemic (49, 87.50%) and reported no family separation (40, 81.63%) as a result of COVID-19. No statistically significant association between professional groups and family separation due to COVID-19 were found, χ^2^(2) = 5.88, *p* = 0.053.

### 3.3. Work Conditions

MHPs reported work changes due to COVID-19 in 50% of cases (*n* = 28), and a worsened relationship with patients due to COVID-19 in 62.50% of cases (*n* = 35).

We detected a statistically significant association between professional groups and changes in the relationship with patients due to COVID-19, *χ^2^*(4) = 11.11, *p* = 0.025. The effect size was small, Cramer’s *V* = 0.182. Post-hoc analysis demonstrated that physicians not working closely with COVID-19 patients were significantly less likely to report worsened relationships with their patients (*z* = −2.81; *p* = 0.045).

### 3.4. Psychological Outcomes

As Table 3 shows, most MHPs reported trait anxiety (37, 66.07%) but no state anxiety (38, 67.86%). The majority of MHPs reported no post-traumatic distress (32, 57.14%), low emotional exhaustion (29, 51.79%), low depersonalization (40, 71.43%), and high personal accomplishment (34, 60.71%).

As Table 4 reports, we found statistically significantly differences in hyperarousal median scores between the different professional groups, *χ^2^*(2) = 19.18, *p* < 0.001. Subsequent pairwise comparisons revealed that hyperarousal scores were statistically significantly lower in MHPs compared to HPs working closely with COVID-19 patients (statistic = −39.45, *p* < 0.001) and statistically significantly higher in HPs working closely with COVID-19 patients compared to HPs not working closely with COVID-19 patients (statistic = 23.90, *p* = 0.027). Moreover, we found significant differences in intrusion median scores between the different professional groups, *χ^2^*(2) = 10.18, *p* = 0.006 (Table 4). Subsequent pairwise comparisons revealed that intrusion scores were statistically significantly lower in MHPs compared to HPs working closely with COVID-19 patients (statistic = −22.76, *p* = 0.036) and to HPs not working closely with COVID-19 patients (statistic = −27.14, *p* = 0.009). We also found statistically significant differences in total post-traumatic median scores between the different professional groups, *χ^2^*(2) = 8.73, *p* = 0.013 (Table 4). Subsequent pairwise comparisons revealed that total post-traumatic distress scores were lower in MHPs compared to HPs working closely with COVID-19 patients (statistic = −26.53, *p* = 0.011).

For the MBI, we found statistically significant differences in depersonalization median scores between the different professional groups, *χ^2^*(2) = 8.25, *p* < 0.001 (Table 4). Subsequent pairwise comparisons revealed that depersonalization scores were lower in MHPs compared to HPs working closely with COVID-19 patients (statistic = −38.27, *p* < 0.001) and HPs not working closely with COVID-19 patients (statistic = −23.22, *p* = 0.034). There was also a statistically significant association between professional groups and depersonalization levels, *χ^2^*(4) = 23.54, *p* < 0.001 (Table 3). This association was moderately strong, Cramer’s *V* = 0.265. Post-hoc analysis demonstrated that MHPs were significantly more likely to report low depersonalization (*z* = 3.99; *p* = 0.001) and less likely to report high depersonalization (*z* = −3.33; *p* = 0.008). Moreover, physicians working closely with COVID-19 patients were less likely to report low depersonalization (*z* = −4.02; *p* = 0.001).

We found statistically significant differences in personal accomplishment between the different professional groups, *χ^2^*(2) = 21.43, *p* < 0.001 (Table 4). Subsequent pairwise comparisons revealed that personal accomplishment scores were higher in MHPs compared to HPs working closely with COVID-19 patients (statistic = 42.08, *p* < 0.001). Moreover, we detected a statistically significant association between professional groups and personal accomplishment levels, *χ^2^*(4) = 22.56, *p* < 0.001 (Table 3). The association was moderately strong, Cramer’s *V* = 0.260. Post-hoc analysis demonstrated that MHPs were more likely to report high personal accomplishment (*z* = 3.86; *p* = 0.001) and less likely to report low personal accomplishment (*z* = −3.74; *p* = 0.002). Conversely, physicians working closely with COVID-19 patients were significantly less likely to report high personal accomplishment (*z* = −3.62; *p* = 0.003) and more likely to report low personal accomplishment (*z* = 3.42; *p* = 0.006).

### 3.5. Factors Associated with Anxiety, Post-Traumatic Stress, and Burnout

Table 5 and Table 6 report the results of the multivariable logistic regressions performed to ascertain the effects of possible risk factors on the likelihood of exhibiting psychological outcomes.

The logistic regression model for state anxiety was statistically significant, *χ*^2^(13) = 65.07, *p* < 0.001. The model explained 48.86% (Nagelkerke *R*^2^) of the variance in state anxiety and correctly classified 75.69% of cases. Its sensitivity was 72.13%, specificity 78.31%, positive predictive value 70.97%, and negative predictive value 79.27%. In particular, MHPs had 0.14 times lower odds of exhibiting state anxiety compared to HPs not working closely with COVID-19 patients (*p* = 0.002); males had 0.17 times lower odds of exhibiting state anxiety compared to females (*p* = 0.002); and professionals with children had 0.29 times lower odds of exhibiting state anxiety compared to professionals without children (*p* = 0.029). Finally, professionals who reported trait anxiety had 23.70 times higher odds of exhibiting state anxiety compared to those who reported no trait anxiety (*p* < 0.001).

The logistic regression model for post-traumatic stress was statistically significant, *χ*^2^(13) = 39.65, *p* < 0.001. The model explained 33.84% (Nagelkerke *R*^2^) of the variance in post-traumatic symptoms and correctly classified 77.08% of cases. Its sensitivity was 51.11%, specificity 88.89%, positive predictive value 67.65%, and negative predictive value 80.00%. In particular, workers whose family separated because of COVID-19 had 4.70 times higher odds of exhibiting post-traumatic symptoms compared to workers whose families did not separate (*p* = 0.002); and professionals who reported trait anxiety had 11.84 times higher odds of exhibiting post-traumatic symptoms compared to those who reported no trait anxiety (*p* = 0.001).

The logistic regression model for emotional exhaustion was statistically significant, *χ*^2^(13) = 29.78, *p* = 0.005. The model explained 25.77% (Nagelkerke *R*^2^) of the variance in emotional exhaustion and correctly classified 72.92% of cases. Its sensitivity was 50.00%, specificity 85.11%, positive predictive value 64.10%, and negative predictive value 76.19%. In particular, professionals with children had 0.31 times lower odds of exhibiting emotional exhaustion compared to professionals without children (*p* = 0.013); and workers who reported extreme changes in their work due to COVID-19 had 4.88 times higher odds of exhibiting emotional exhaustion compared to workers who reported no changes (*p* = 0.044). Finally, professionals who reported trait anxiety had 6.27 times higher odds of exhibiting emotional exhaustion compared to those who reported no trait anxiety (*p* < 0.001).

The logistic regression model for depersonalization was statistically significant, *χ*^2^(13) = 30.63, *p* = 0.004. The model explained 29.33% (Nagelkerke *R*^2^) of the variance in depersonalization and correctly classified 77.78% of cases. Its sensitivity was 21.88%, specificity 93.75%, positive predictive value 50.00%, and negative predictive value 80.77%. In particular, participants who lived separated from their families during the pandemic had 2.89 times higher odds to exhibit depersonalization compared to those whose families did not separate (*p* = 0.052) and professionals who reported trait anxiety had 5.62 times higher odds to exhibit depersonalization compared to those who reported no trait anxiety (*p* = 0.010).

The logistic regression model for personal accomplishment was statistically significant, *χ*^2^(13) = 30.35, *p* = 0.004. The model explained 26.60% (Nagelkerke *R*^2^) of the variance in personal accomplishment and correctly classified 70.81% of cases. Its sensitivity was 41.30%, specificity 84.69%, positive predictive value 55.88%, and negative predictive value 75.45%. In particular, MHPs had 0.30 times lower odds of exhibiting low personal accomplishment compared to HPs not working closely with COVID-19 patients (*p* = 0.041); moreover, professionals who reported trait anxiety had 2.86 times higher odds of exhibiting low personal accomplishment compared to those who reported no trait anxiety (*p* = 0.031).

## 4. Discussion

This study investigated anxiety, post-traumatic distress, and burnout in a sample of MHPs, comparing their distress to that experienced by other HPs, and focusing on potential sociodemographic, family, and occupational factors.

Most MHPs were female and aged ≥41 years. They mostly reported having children, not living alone before the pandemic, and not experiencing family separation as a result of COVID-19. Consistent with the literature [48,49], they also reported considerable or extreme work changes due to COVID-19 in 50% of cases, and a worsened relationship with patients due to COVID-19 in 62.50% of cases. Indeed, COVID-19 patients need both comprehensive and specific medical treatment, so the outbreak brings great challenges to HCWs. This may have worsened the relationship between HCWs and their patients. When the survey was administered, care for COVID-19 patients was primarily medical, without the integration of psychological care. It is, therefore, unsurprising that HCWs who did not work closely with COVID-19 patients were significantly less likely to report worsened relationships with patients compared to professionals working closely with COVID-19 patients. Indeed, previous research has pointed out that during the COVID-19 emergency, establishing a functional relationship is difficult for both clinicians and COVID-19 patients, yet good communication can help patients share their burden and recognize that they are not suffering alone [50]. However, even if MHPs did not work with COVID-19 patients, they did not show differences in the perceived changes in their relationship with their patients compared to physicians working closely with COVID-19 patients. This could be explained by most MHPs working remotely during the first phase of the pandemic, representing a significant change in their clinical setting. Indeed, some sensory and bodily characteristics of the face-to-face psychological and psychotherapeutic setting cannot be fully replicated in an online setting [51,52]. In particular, we should consider the impact of virtually getting into patients’ houses and letting patients into one’s own home, the slight delay in feedbacks from facial microexpressions and the sound of the patient’s voice during videocalls, the different visual perspective, and the different interpersonal distance from patients.

Regarding psychological outcomes, MHPs reported good overall mental health except for trait anxiety. In this context, previous research has shown that traumatic experiences can induce changes in trait variables [53], particularly in trait anxiety [54,55]. Thus, the high trait anxiety in our sample could be interpreted as an effect of the pandemic itself.

Most MHPs reported no post-traumatic distress, and their post-traumatic symptom scores were statistically significantly lower compared to HPs working closely with COVID-19 patients. Meanwhile, hyperarousal scores were statistically significantly lower in MHPs compared to HPs working closely with COVID-19 patients, whose scores were also statistically significantly higher compared to those not working closely with COVID-19 patients. Moreover, intrusion scores were statistically significantly lower in MHPs compared to all other professionals. Regarding MBI scores, MHPs reported low emotional exhaustion in 51.79% of cases, low depersonalization in 71.43% of cases, and high personal accomplishment in 60.71% of cases. In particular, contrasting with previous research [32], depersonalization scores were statistically significantly lower in MHPs compared to all other HPs, while personal accomplishment scores were statistically significantly higher in MHPs compared to HPs working closely with COVID-19 patients. Moreover, multivariable logistic regressions showed that MHPs had lower odds of exhibiting state anxiety and low personal accomplishment compared to HPs not working closely with COVID-19 patients.

It seems that MHPs can rely on good affective regulation strategies, allowing them to be more emotionally responsive and cognitively present in clinical practice. In other words, it seems that a mind trained to think as if bombs are falling down should be a fundamental affective resource when the bombs are, in fact, falling down. This invites reflection on the importance of MHPs’ personal and professional training and on the specific impact on MHPs’ mental functioning of work in which steps are not connected to concrete, objective data. Thus, MHPs can be a key resource in health organizations’ decision-making processes regarding patients’ care [56,57]. However, we must keep in mind that during the first phase of the pandemic, most MHPs did not work with COVID-19 patients: thus, we have to consider and implement policies aimed at preserving MHPs’ mental health. Indeed, though not every professional is needed at the forefront of managing a pandemic, it is vital to preserve a field in which different psychological functions may express and evolve.

As expected, professionals who reported trait anxiety had higher odds of exhibiting state anxiety, post-traumatic symptoms, emotional exhaustion, depersonalization, and low personal accomplishment compared to professionals who reported no trait anxiety. Previous literature suggests that anxiety can accentuate post-traumatic stress reactions: individuals with trait anxiety may respond more extremely to a traumatic stressor, distressed by not only the event but also their own reactions [58,59].

Family separation was a significant predictor for post-traumatic symptoms and depersonalization, consistently with previous research suggesting that physical separation or infrequent communication were important sources of distress for HCWs and their families [60,61]. Gender was a significant predictor only for state anxiety. The role of gender with respect to anxiety has already been widely investigated: several studies have shown that women are at greater risk of developing mental health problems, especially anxiety and depression, during the COVID-19 pandemic [20,62,63]. Moreover, contrary to previous research [64,65], professionals with children reported lower odds of exhibiting state anxiety and emotional exhaustion compared to professionals without children. It seems that the potential distress connected to taking care of children, particularly with homeschooling and changes in daily routines, is lower than the emotional benefits of the parent–child bond. It would be interesting to explore if this association is influenced by child age, romantic relationship with the partner, and affects shared within the family.

Finally, workers who reported an improved relationship with patients due to COVID-19 showed higher odds of exhibiting state anxiety compared to those who reported no change in this relationship. It is plausible that those professionals able to describe changes in the quality of their affective bond with patients are also more aware of their own emotional responses, including anxiety.

The health emergency we are experiencing due to the spread of COVID-19 has strongly influenced the psychological and physical health of the general population, including that of HPs. As the mental healthcare demand is especially high during these times and MHPs have to shoulder this responsibility, there is potential for their stress to increase, with adverse effects on their own mental health. However, our data suggest that MHPs’ mental health has been almost preserved in the first phase of the pandemic, and it would be useful to rely on these professionals going forward, especially for structuring interventions to improve and support the mental health of the general population and of other HCWs.

### Limitations and Future Directions

This study has some critical limitations. First, the generalizability of results is limited by our small sample derived from one local health authority in North Italy. It would be interesting to compare our results to those detected in other healthcare systems or territories with a different pandemic situation. Second, the cross-sectional design does not allow for causal inferences, so we should be cautious of interpreting the findings as evidence of predictive links between the studied variables. Longitudinal studies are needed to explore the development of distress in HPs—particularly in MHPs—over time and its association with other clinical, family, and occupational variables. Finally, psychological variables were assessed through self-report measures, so further studies should also consider clinical and observational data.

## 5. Conclusions

Despite its limitations, this study is the first attempt to reveal the impact of the COVID-19 pandemic on MHPs, focusing on state and trait anxiety, post-traumatic stress, and burnout, and on their connections with sociodemographic, family, and occupational factors. Moreover, our data elucidate differences in the impact experienced by MHPs and other HCWs. The emotional distress faced by HCWs during a pandemic is a key public concern. Our results suggest that MHPs have adequate resources to handle the impact of the pandemic, and can, thus, be useful in not only helping patients and their families facing the disease but also supporting other health professional, reducing their emotional burden and helping them provide better care to patients.

## Figures and Tables

**Table 1:** Study participants.

	n	%
Mental health professionals		
Psychiatrists	16	28.57
Psychologists	40	71.43
Total	56	100.00
Physicians working closely with COVID-19 patients		
Anesthesiologists	28	49.12
Infectious diseases specialists	15	26.32
ED physicians	14	24.56
Total	57	100.00
Physicians not working closely with COVID-19 patients		
Pathologists	2	3.70
General Surgeons	3	5.56
Endocrinologists	7	12.96
Gynecologists	4	7.41
Preventive medicine specialists	8	14.81
Forensic pathology physicians	4	7.41
Ophthalmologists	2	3.70
Dentists	3	5.56
Oncologists	2	3.70
Radiologists	4	7.41
Urologists	2	3.70
Other physicians *	13	24.07
Total	54	100.00

* E.g., occupational health physicians, rehabilitation physicians, neurosurgeons, orthopedics, clinical pathologists, nutritionists.

**Table 2:** Contact with COVID-19 patients.

	Mental Health Professionals (*n* = 56)		Physicians Working Closely with COVID-19 Patients (*n* = 57)		Physicians not Working Closely with COVID-19 Patients (*n* = 54)	
	*n*	%	*n*	%	*n*	%
Contact with COVID-19 patients in the last two months						
None	47	83.93	0	0.00	24	44.44
Rare	9	16.07	0	0.00	30	55.56
Frequent	0	0.00	57	100.00	0	0.00

**Table 3:** Differences between categorical variables.

	Mental Health Professionals (*n* = 56)		Physicians Working Closely with COVID-19 Patients (*n* = 57)		Physicians not Working Closely with COVID-19 Patients (*n* = 54)				
	*n*	%	*n*	%	*n*	%	*χ2*	*df*	*p*
Socio-Demographic Characteristics									
Age range							43.52	4	<0.001
≤40 years	6	10.71	29	50.88	7	12.96			
41-50 years	27	48.21	16	28.07	11	20.37			
≥50 years	23	41.07	12	21.05	36	66.67			
Gender							2.54	2	0.282
Female	48	85.71	38	66.67	31	57.41			
Male	8	14.29	19	33.33	23	42.59			
Family Conditions									
Children							2.54	2	0.282
No	19	33.93	24	42.11	15	27.78			
Yes	37	66.07	33	57.89	39	72.22			
Living alone before the pandemic							2.47	2	0.291
No	49	87.50	46	80.70	49	90.74			
Yes	7	12.50	11	19.30	5	9.26			
Family separation due to COVID-19							5.88	2	0.053
No	40	81.63	29	63.04	40	81.63			
Yes	9	18.37	17	36.96	9	18.37			
Working Conditions									
Work changes due to COVID-19							0.47	6	0.998
Not at all	14	25.00	13	22.81	15	27.78			
Slightly	14	25.00	16	28.07	14	25.93			
Considerably	20	35.71	20	35.09	18	33.33			
Extremely	8	14.29	8	14.04	7	12.96			
Changes in the relationship with patients due to COVID-19							11.11	4	0.025
Not at all	7	12.50	11	19.30	12	22.22			
Improved	14	25.00	7	12.28	19	35.19			
Worsened	35	62.50	39	68.42	23	42.59			
Psychological Distress									
STAI-Y Trait Anxiety							0.78	2	0.676
No trait anxiety	19	33.93	15	26.32	16	29.63			
Trait anxiety	37	66.07	42	73.68	38	70.37			
STAI-Y State Anxiety							5.63	2	0.060
No state anxiety	38	67.86	27	47.37	27	50.00			
State anxiety	18	32.14	30	52.63	27	50.00			
IES-R TOT							11.68	6	0.069
Normal	32	57.14	24	42.11	31	57.41			
Mild	13	23.21	8	14.04	5	9.26			
Moderate	2	3.57	5	8.77	6	11.11			
Severe	9	16.07	20	35.09	12	22.22			
MBI Emotional Exhaustion							4.87	4	0.301
Low	29	51.79	20	35.09	20	37.04			
Medium	11	19.64	16	28.07	11	20.37			
High	16	28.57	21	36.84	23	42.59			
MBI Depersonalization							23.54	4	<0.001
Low	40	71.43	16	28.07	27	50.00			
Medium	11	19.64	21	36.84	11	20.37			
High	5	8.93	20	35.09	16	29.63			
MBI Personal Accomplishment							22.56	4	<0.001
Low	8	14.29	29	50.88	19	35.19			
Medium	14	25.00	16	28.07	14	25.93			
High	34	60.71	12	21.05	21	38.89			

**Table 4:** Differences between continuous variables.

	Mental Health Professionals (*n* = 56)		Physicians Working Closely with COVID-19 Patients (*n* = 57)		Physicians not Working Closely with COVID-19 Patients (*n* = 54)				
							Kruskal–Wallis Test		
	Med	IQR	Med	IQR	Med	IQR	*χ^2^*	df	*p*
STAI-Y									
Trait Anxiety	43.00	13.75	50.00	19.00	47.00	20.25	4.26	2	0.119
State Anxiety	34.00	12.75	40.00	13.50	39.50	13.25	5.68	2	0.058
IES-R									
Hyperarousal	0.86	0.96	1.43	1.28	1.00	0.72	19.18	2	<0.001
Avoidance	0.50	0.75	0.75	0.88	0.75	1.50	2.55	2	0.280
Intrusion	1.00	0.94	1.75	1.25	1.50	1.50	10.18	2	0.006
TOT	18.00	17.50	29.00	23.00	22.50	21.00	8.73	2	0.013
MBI									
Emotional exhaustion	13.00	17.00	20.00	24.00	19.00	23.00	4.44	2	0.109
Depersonalization	2.00	4.00	7.00	7.00	3.50	8.00	18.14	2	<0.001
Personal accomplishment	39.00	10.00	29.00	14.00	34.50	44.00	21.43	2	<0.001

**Table 5:** Multivariable logistic regressions on state anxiety and post-traumatic stress.

	STAI-Y State Anxiety		IES-R TOT	
	OR (95% CI)	*p*	OR (95% CI)	*p*
Socio-Demographic Characteristics				
Group				
Physicians not working closely with COVID-19 patients	Ref		Ref	
Physicians working closely with COVID-19 patients	0.46 (0.13–1.66)	0.238	0.96 (0.30–3.05)	0.984
Mental health professionals	0.14 (0.04–0.49)	0.002	0.43 (0.14–1.30)	0.134
Age range				
≤40 years	Ref		Ref	
41-50 years	2.47 (0.67–9.10)	0.174	3.07 (0.89–10.54)	0.075
≥50 years	0.67 (0.18–2.55)	0.558	1.24 (0.35–4.41)	0.741
Gender				
Female	Ref		Ref	
Male	0.17 (0.06–0.52)	0.002	0.51 (0.19–1.38)	0.185
Family Conditions				
Children				
No	Ref		Ref	
Yes	0.29 (0.09–0.88)	0.029	1.58 (0.57–4.43)	0.381
Family separation due to COVID-19				
No	Ref		Ref	
Yes	1.16 (0.40–3.40)	0.785	4.70 (1.73–12.82)	0.002
Working Conditions				
Work changes due to COVID-19				
Not at all	Ref		Ref	
Slightly	0.49 (0.14–1.75)	0.275	0.79 (0.24–2.62)	0.695
Considerably	1.11 (0.35–3.55)	0.860	1.02 (0.34–3.07)	0.972
Extremely	0.50 (0.09–2.92)	0.442	1.18 (0.22–6.24)	0.849
Changes in the relationship with patients due to COVID-19				
Not at all	Ref		Ref	
Improved	3.27 (0.76–13.96)	0.110	0.70 (0.19–2.63)	0.600
Worsened	1.32 (0.37–4.71)	0.673	0.73 (0.23–2.33)	0.597
Psychological Characteristics				
STAI-Y Trait Anxiety				
No	Ref		Ref	
Yes	23.70 (6.46–86.93)	<0.001	11.84 (3.07–45.62)	<0.001

**Table 6:** Multivariable logistic regressions on burnout.

	MBI Emotional Exhaustion		MBI Depersonalization		MBI Personal Accomplishment	
	OR (95% CI)	*p*	OR (95% CI)	*p*	OR (95% CI)	*p*
Socio-Demographic characteristics						
Group						
Physicians not working closely with COVID-19 patients	Ref		Ref		Ref	
Physicians working closely with COVID-19 patients	0.43 (0.14–1.31)	0.138	0.53 (0.16–1.80)	0.312	1.49 (0.53–4.18)	0.452
Mental health professionals	0.39 (0.14–1.12)	0.080	0.33 (0.09–1.20)	0.093	0.30 (0.09–0.95)	0.041
Age range						
≤40 years	Ref		Ref		Ref	
41–50 years	2.17 (0.68–6.89)	0.188	0.33 (0.09–1.19)	0.090	0.39 (0.12–1.12)	0.108
≥50 years	1.28 (0.40–4.15)	0.678	0.35 (0.10–1.24)	0.104	0.69 (0.23–2.13)	0.521
Gender						
Female	Ref		Ref		Ref	
Male	0.90 (0.36–2.25)	0.825	1.78 (0.65–4.90)	0.265	1.10 (0.45–2.71)	0.828
Family Conditions						
Children						
No	Ref		Ref		Ref	
Yes	0.31 (0.12–0.78)	0.013	0.41 (0.15–1.12)	0.080	0.62 (0.24–1.58)	0.318
Family separation due to COVID-19						
No	Ref		Ref		Ref	
Yes	1.30 (0.51–3.36)	.584	2.89 (0.99–8.40)	0.052	1.63 (0.62–4.27)	0.320
Working Conditions						
Work changes due to COVID-19						
Not at all	Ref		Ref		Ref	
Slightly	2.36 (0.71–7.79)	0.159	1.61 (0.40–6.58)	0.505	2.02 (0.63–6.42)	0.234
Considerably	2.40 (0.80–7.20)	0.117	1.50 (0.43–5.28)	0.527	0.80 (0.26–2.42)	0.690
Extremely	4.88 (1.04–22.79)	0.044	0.92 (0.14–5.89)	0.930	2.18 (0.48–9.95)	0.315
Changes in the relationship with patients due to COVID-19						
Not at all	Ref		Ref		Ref	
Improved	0.74 (0.21–2.70)	0.654	0.39 (0.09–1.72)	0.214	0.87 (0.24–3.15)	0.827
Worsened	1.07 (0.34–3.31)	0.912	0.57 (0.16–2.04)	0.388	0.82 (0.26–2.54)	0.728
PSychological Characteristics						
STAI-Y Trait Anxiety						
No	Ref		Ref		Ref	
Yes	6.27 (2.23–17.58)	<0.001	5.62 (1.51–20.89)	0.010	2.86 (1.10–7.44)	0.031

## Data Availability

The datasets generated for this study are available on request from the corresponding author.
